# Quality of biological images, reconstructed using localization microscopy data

**DOI:** 10.1093/bioinformatics/btx597

**Published:** 2017-09-25

**Authors:** Błażej Ruszczycki, Tytus Bernas

**Affiliations:** Nencki Institute of Experimental Biology, Center of Neurobiology, Polish Academy of Sciences, ul. Pasteura 3, Warsaw, Poland

## Abstract

**Motivation:**

Fluorescence localization microscopy is extensively used to study the details of spatial architecture of subcellular compartments. This modality relies on determination of spatial positions of fluorophores, labeling an extended biological structure, with precision exceeding the diffraction limit. Several established models describe influence of pixel size, signal-to-noise ratio and optical resolution on the localization precision. The labeling density has been also recognized as important factor affecting reconstruction fidelity of the imaged biological structure. However, quantitative data on combined influence of sampling and localization errors on the fidelity of reconstruction are scarce. It should be noted that processing localization microscopy data is similar to reconstruction of a continuous (extended) non-periodic signal from a non-uniform, noisy point samples. In two dimensions the problem may be formulated within the framework of matrix completion. However, no systematic approach has been adopted in microscopy, where images are typically rendered by representing localized molecules with Gaussian distributions (widths determined by localization precision).

**Results:**

We analyze the process of two-dimensional reconstruction of extended biological structures as a function of the density of registered emitters, localization precision and the area occupied by the rendered localized molecule. We quantify overall reconstruction fidelity with different established image similarity measures. Furthermore, we analyze the recovered similarity measure in the frequency space for different reconstruction protocols. We compare the cut-off frequency to the limiting sampling frequency, as determined by labeling density.

**Availability and implementation:**

The source code used in the simulations along with test images is available at https://github.com/blazi13/qbioimages.

**Supplementary information:**

Supplementary data are available at *Bioinformatics* online.

## 1 Introduction

Fluorescence optical microscopy is extensively used to gain insight into spatial distribution and behavior of biomolecules in cellular context. Information about the components of cell architecture, influenced by processes occurring at the spatial scale of few nanometers, is limited by diffraction of light. A typical diameter of diffraction image of a point source in focus of a high NA (∼1.4) microscope objective corresponds to 200 nm or more (depending on a wavelength). On the other hand, the position of such a point source can be localized with precision exceeding the diffraction limit (Henriques and Mhlanga, 2009; Henriques *et al.*, 2011; Yildiz *et al.*, 2003). This mode of detection is employed by super-resolution microscopy based single-molecule localization, developed independently by several groups and named STORM (Rust *et al.*, 2006), PALM (Betzig *et al.*, 2006) and FPALM (Hess *et al.*, 2006). The basic principle is to use photoswitchable fluorescent probes, activate and image simultaneously only a small, optically resolvable fraction of fluorophores. With repetitive imaging cycles, the positions of all fluorophores in the sample are determined, allowing for reconstruction of a super-resolution image. Numerous photoactivatable fluorescent proteins and organic fluorophores have been created in the past few years to address this need (Fernández-Suárez and Ting, 2008; Folling *et al.*, 2007; Lukyanov *et al.*, 2005). Moreover, the possibility of optical switching of numerous traditional fluorescent labels, already established in biological microscopy, has been demonstrated (Heilemann *et al.*, 2009; van de Linde *et al.*, 2009). One should note that a STORM/PALM system is essentially similar in configuration to a wide-field fluorescence microscope (Cebecauer *et al.*, 2012; Henriques *et al.*, 2011). Therefore, its inherently high light throughput (due to simplicity of a design) may be combined with efficient array detectors (EMCCD or sCMOS). Owing to these advantages, localization precision of 20 nm may be obtained in biological imaging (Bates *et al.*, 2007; Dempsey *et al.*, 2011). Several established models describe influence of pixel size, signal-to-noise ratio and optical resolution on the precision available in a given experimental system (Ober *et al.* 2004; Ram *et al.*, 2006; Thompson *et al.*, 2002). The labeling density has been also recognized as an important factor affecting reconstruction fidelity of imaged biological structures (Huang *et al.*, 2009; Sinko *et al.*, 2014; van de Linde *et al.*, 2011). Likewise, image quality measures, based on FRC (Fourier Ring Correlation), have been developed specifically for localization microscopy (Banterle *et al.*, 2013; Nieuwenhuizen *et al.*, 2013).

Nonetheless, quantitative results on this subject are scarce. It should be noted that processing STORM/PALM microscopy data is similar reconstruction of a continuous (extended) non-periodic signal from a non-uniform noisy point samples (Kazakov, 2006; Strohmer and Tanner, 2006). Typically, such tasks are realized with numeric optimization, as few analytical solutions exist (Kazakov, 2006). In two dimensions, the problem may be formulated within framework of matrix completion (Candes and Plan, 2010; Candes and Tao, 2010). However, no systematic approach has been adopted in microscopy, where images are usually rendered by representing localized molecules with Gaussian distributions (widths determined by localization precision).

Here, we analyze the process of two-dimensional reconstruction of extended biological structures as a function of the density of registered emitters, localization precision (which is affected by signal-to-noise ratio) and the area occupied by the rendered localized molecule. In order to assess the reconstruction fidelity, we take electron microscopy images as the ground truth. The image defines the local emitter (labeling) density and serves as the reference for reconstruction simulations. We quantify overall reconstruction fidelity with different established image similarity measures. Furthermore, we analyze the recovered spatial frequency spectrum (we study similarity norm in the frequency space) for different reconstruction arrangements. A similar approach was presented in other studies (Nieuwenhuizen *et al.*, 2013) where a frequency measure (FRC) was used to determine a resolution of a single image (dividing a set of single-emitter localization into two statistically independent subsets and removing spurious correlations resulting from repeated activation of the same emitters). Conversely, in our approach we use an equivalent measure in the frequency space to compare the quality of reconstructed image with the ground truth (reference), which corresponds to continuous emitter density. With this setup, we express an image quality *IQ* (by calculating scalar similarity measures, or a frequency space similarity measure) as a function of both average emitters density *N*_avg_, localization accuracy κ and rendered area size *d*, i.e.

$$IQ=IQ(N_{\text{avg}},\kappa ,d)$$

The localization accuracy is in turn a function of other parameters such as total photon number *n*_photon_, camera noise ρ, observation wavelength λ, type of reconstruction algorithm *k*_alg_; the image quality *IQ* depends on these parameters implicitly via $\kappa =\kappa (n_{\text{photon}},\rho ,\lambda ,k_{alg})$. This relation has been extensively studied by others (see Ober *et al.*, 2004; Ram *et al.*, 2006; Thompson *et al.*, 2002). Hence, we present the results as a function of localization accuracy κ, rather as a function of underlying parameters in order to reduce the dimension of parameter space, simplifying interpretation of results.

## 2 Background

### 2.1 Quantitative measures of image similarity

Since our main objective is to analyze the fidelity of the image reconstruction, we first need to define the quantitative measures which will be used to compare the reconstructed image with the reference image. These measures should: (i) be independent on average image intensities (as there is no natural overall image scale for the reconstructed image) and (ii) have simple interpretation and construction. Several image similarity (quality) measures have been proposed in area of microscopy (Di Gesù and Starovoitov, 1999; Goshtasby, 2012; Khazenie *et al.*, 1992; Mitchell, 2010; Sage *et al.*, 2015; Sinha and Russell, 2011; Thung and Raveendran, 2010; Wang and Bovik, 2006). However, not all are adequate for our purposes. These include SSIM (compares average image intensities), PNSR (sensitive to image maximum and overall reconstruction intensity), measures based on Mutual Information (require grayscale continuous model, for images with sparse reconstruction we have only grayscale bands), ranking measures (problematic in the presence of ties, produced by small size of emitter sets). Therefore, for our analysis we use the following measures:

**A.** Pearson Correlation Coefficient.

The most common measure of images similarity (in case when there is no coordinate transformation, such as a shift, rotation, scaling or distortion) between two images, is the Pearson correlation coefficient defined as:

(1) $$Q_{\text{pearson}}=\frac{\sum_{x,y}((I_{0}(x,y)-{\bar{I}}_{0})(I_{\text{rec}}(x,y)-{\bar{I}}_{\text{rec}})}{\sqrt{\sum_{x,y}((I_{0}(x,y)-{\bar{I}}_{0}{)}^{2}\sum_{x,y}(I_{\text{rec}}(x,y)-{\bar{I}}_{\text{rec}}{)}^{2}}},$$

where $I_{0}(x,y)$ and $I_{\text{rec}}(x,y)$ denote pixel intensity of the reference image and the reconstructed image, respectively. ${\bar{I}}_{0},\;{\bar{I}}_{\text{rec}}$ denote the average pixel intensities for both images.

**B.** Agreement Between Binarized Images.

Quite often, the observed images are binarized (thresholded) in order to extract and to measure the structure of interest. We study such cases, comparing the overlap between the binarized reference and reconstructed images, denoted as $I_{0}^{\text{bin}}$ and $I_{\text{rec}}^{\text{bin}}$ respectively. We determine the threshold by Otsu method (Otsu, 1979). The agreement between binarized images is defined as:

(2) $$Q_{\text{binarized}}=1-\frac{\sum_{x,y}|I_{0}^{\text{bin}}(x,y)-I_{\text{rec}}^{\text{bin}}(x,y)|}{\sum_{x,y}I_{0}^{\text{bin}}(x,y)}.$$

**C.** Normalized Square *L*_2_ Norm. This measure is defined as:

(3) $$Q_{L_{2}}=\sum_{x,y}{\left(\frac{I_{0}(x,y)-{\bar{I}}_{0}}{\sqrt{\sum_{x,y}{\left(I_{0}(x,y)-{\bar{I}}_{0}\right)}^{2}}}-\frac{I_{\mathrm{rec}}(x,y)-{\bar{I}}_{\mathrm{rec}}}{\sqrt{\sum_{x,y}{\left(I_{\mathrm{rec}}(x,y)-{\bar{I}}_{\mathrm{rec}}\right)}^{2}}}\right)}^{2}.$$

It is insensitive to image contrast and emphasizes larger intensity differences between the analyzed images (Goshtasby, 2012). For the perfect agreement of the images the value of this norm is zero.

**D.** Similarity Measure in Frequency Space.

**Fig. 1.**
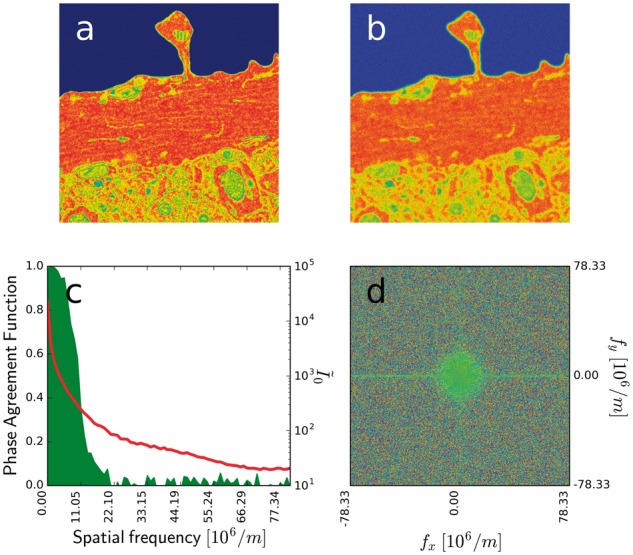
Similarity measure in frequency space. (**a**) Test image; (**b**) Test image after Gaussian blur with added noise; (**c**) Similarity measure $Q_{\text{freq}}(f)$ in frequency space (shaded green area, left scale), frequency spectrum (red line, right scale). (**d**) Phase agreement map between the two images, $\eta_{0}(f_{x},f_{y})\eta_{\text{rec}}^{*}(f_{x},f_{y})$

So far, we discussed only the scalar measures, that do not provide information about reconstruction quality at different spatial scales. Therefore, we introduce a function $Q_{\text{freq}}(f)$ that takes value 1 if at a given frequency there is a perfect agreement between the images, and 0 if at a given frequency the images are uncorrelated. We construct such a function in the following way: Let us denote by $\hat{\hat{I_{0}}}$ and $\hat{\hat{I_{\text{rec}}}}$ the Fourier transforms of the reference and the reconstructed image, respectively. We decompose both these quantities into the pure phase and the amplitude factors, i.e.

(4) $$\hat{\hat{I_{0}}}(f_{x},f_{y})=e^{i\eta_{0}(f_{x},f_{y})}A_{0}(f_{x},f_{y}),$$

(5) $$\hat{\hat{I_{\text{rec}}}}(f_{x},f_{y})=e^{i\eta_{\text{rec}}(f_{x},f_{y})}A_{\text{rec}}(f_{x},f_{y}),$$

where $A_{0}(f_{x},f_{y}),A_{\text{rec}}(f_{x},f_{y})\in \mathbb{R}$. We define

(6) $$Q_{\text{freq}}(f)=\sum_{\begin{array}{c} f_{x},f_{y}\\ f^{2}<f_{x}^{2}+f_{y}^{2}\leq {\left(f+\delta_{f}\right)}^{2} \end{array}}\eta_{0}(f_{x},f_{y})\eta_{\text{rec}}^{*}(f_{x},f_{y}),$$

i.e. we average the phase agreement within the ring $]f,f+\delta_{f}]$. Here, δ_*f*_ defines us the resolution of the $Q_{\text{freq}}(f)$ function (in practice, it is determined by the number of finite sampling *k* of the *f* domain, as $\delta_{f}=f_{\text{max}}/k$). Figure 1 shows an example of the constructed measure, which is mathematically similar to FRC. The test image was modified to distort the high frequency content by blurring and adding the noise. The phase agreement map (panel d) shows the term in the sum in (8), panel c shows the constructed measure. 

### 2.2 Simulations

The goal of the simulations is to obtain the reconstructed STORM image, with the assumed emitters density ρ(x,y), normalized to the interval [0,1]. The procedure is performed as follows: We define *N*_max_, the maximal number of available emitters. We randomly pick the emitter coordinates *x*, *y* and a random number *p* from the interval [0, 1]. When p≤ρ(x,y), we append the emitter with the coordinates $x+\delta_{x},y+\delta_{y}$ to the list, where δ_*x*_ and δ_*y*_ are drawn from the Gaussian distribution with 0 mean and δ_*κ*_ deviation. The latter parameter represents accuracy in determining the emitter coordinates, which is affected by the optical resolution, pixel size and the photon noise. We repeat the procedure until we reach the desired number of emitters *N*_max_. Subsequently, we start reconstruction of the image, recording the intermediate frames. In each step we select randomly *N_f_* emitters from the emitter list, and depending on the selected configuration we add a *d *×* d* square, or the Gaussian distribution with deviation of *d* at the positions of each selected emitter. These emitters are subsequently removed from the list. The procedure is continued until we use all available emitters. For each intermediate frame we calculate and record the values of $Q_{\text{pearson}},Q_{\text{binarized}},Q_{L_{2}}$ and $Q_{\text{freq}}(f)$ quantities.

### 2.3 Distribution of local sampling densities and limiting precision

We estimate local density of spatial sampling (a limiting factor in the reconstruction) by quantifying spacing between nearest localized emitters. This topic has been already extensively studied in the context of localization microscopy (see Baddeley *et al.*, 2010; Deschout *et al.*, 2014; Dylan *et al.*, 2013; Levet *et al.*, 2015). The images, reconstructed with the *N_f_* emitters, are rendered with maximum accuracy (using a single pixel for each emitter). The area corresponding to an emitter is calculated using Voronoi tesselation, as described in Jones *et al.* (2005). The sampling interval is then set as the radius (δ_*r*_) of the equivalent area circle. The limiting precision (δ_*l*_) is calculated as:

(7) $$\delta_{l}=\sqrt{\delta_{r}^{2}+\delta_{g}^{2}}.$$

The corresponding limiting frequency was $\delta_{l}/2$.

### 2.4 Sample preparation and imaging

Slices of rat dentate gyrus brain region were fixed in 3% glutaraldehyde and dehydrated as described in Rouquette *et al.* (2009). DNA was labeled by a pre-embedding method based on the NAMA-Ur procedure (Testillano *et al.*, 1991). Slices of rat hippocampal CA1 brain region were fixed in 2% formaldehyde and 2.5% glutaraldehyde as described in Wilke *et al.* (2013). The fixed materials were labeled with osmium tetroxide, uranyl acetate and lead aspartate using the procedure described before (Walton, 1979). The brain tissue was imaged using a SEM microscope (Sigma VP, Zeiss), equipped with an automated and a BSD detector (3View, Gattan Inc.). The images were registered at 5–6 kEV and 15 000× magnification in the low vacuum mode (2–5 Pa) and with 16 bit precision. The frame size was 2048 × 2048 pixels (6.4 nm pixel size) and the section thickness 50 nm.

## 3 Results and discussion

**Fig. 2.**
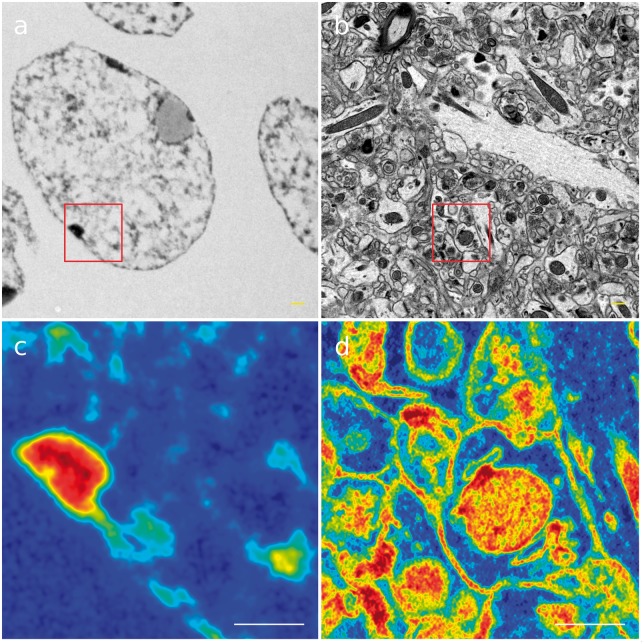
Reference electron-microscopy images used in simulations: (**a**) cellular nucleus; (**b**) neuronal tissue; (**c**) and (**d**) magnification of regions of interest in pseudocolor scale (entire images were used in simulations); scale bars 600 nm

In order to assess the quality of the image reconstruction, we performed simulations for different values of emitter density, emitter localization accuracy, size of rendered area of a single emitter, type of rendered area (square or Gaussian), for two different reference images (NT—neuronal tissue and CN—cellular nuclei, see Fig. 2). For each of the simulation setting we calculated the values of the parameters (2), (3), (5) and (8) for the entire images (see Fig. 2), however hereafter we show only the reconstruction of the regions of interest (ROIs) to emphasis the image details. From now on, all the presented figures refer to Image NT (neuronal tissue). The numerical results and the reconstructed ROI for Image CN (cellular nucleus) are provided in the Supplementary Material. 

**Fig. 3.**
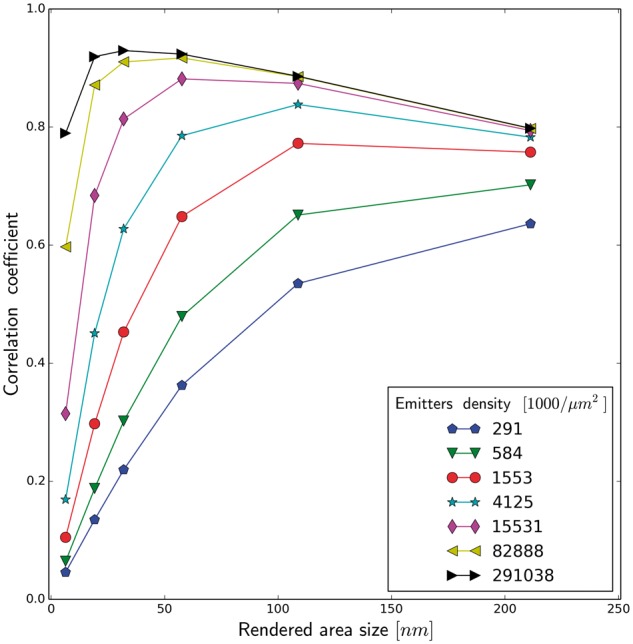
Correlation coefficient as a function of rendered area size (side of square rendering) for different emitters density, Image II (neuronal tissue), emitter localization accuracy 25 nm

**Fig. 4.**
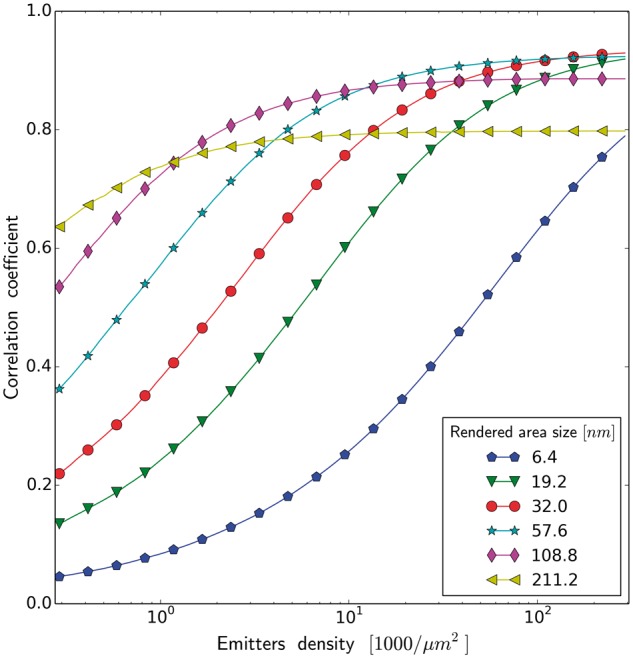
Correlation coefficient as a function of emitters density for different rendered area size (side of square rendering), (image of neuronal tissue), emitter localization accuracy 25 nm

**Fig. 5.**
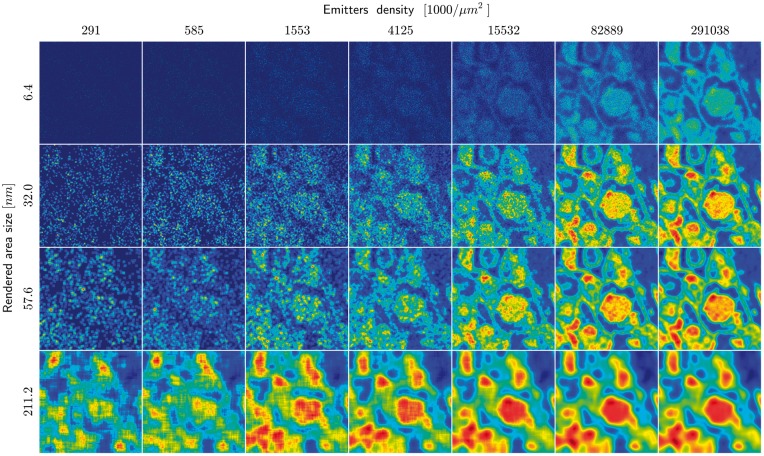
Reconstruction of the ROI for different rendering square size and emitter density (image of neuronal tissue), emitter localization accuracy 25 nm

In the first place, we analyze the reconstruction quality as a function of an area assigned to the single localized emitter. As each localization corresponds to local density of molecules, to simplify the analysis, we associate the localization with a finite and bound area of standardized shape. Therefore, we render the molecules position with an area of square pixels. Later, we compare to obtained results with a rendering with Gaussian functions (see Supplementary Material). Figure 3 shows the correlation coefficient as the function of emitters density in range $291-291\;000\;\text{per}\;{\mu m}^{2}$ (which corresponds to $50\cdot 10^{3}-50\cdot 10^{6}$ per image), for different values of *d*, the size of side of the squares used to render the emitters (*d*) in the reconstructed image. We observe immediately that the best rendering (measured by the value of the correlation coefficient) for the small number of emitters is achieved by using the largest squares, i.e. *d* = 211.2 nm. As we increase the numbers of emitters, the plotted functions cross each other. When we surpass the emitters density ∼1150 per μm^2^ the highest correlation coefficient is achieved for the squares with *d* = 108.8 nm, and so on. Even for the extremely large number of emitters ($50\times 10^{6}$) per image, the rendering with the smallest squares *d* = 6.4 nm yields the value of the correlation coefficient substantially smaller, than the rendering with the optimal square, here *d* = 32 nm. On the other hand we observe, that for the functions with the large squares (*d* = 211.2 nm and *d* = 108.8 nm) and after collecting a certain number of emitters no further increase of correlation coefficient is obtained. Thus, increase of the number of emitters does not improve fidelity of reconstruction of the imaged biological structure. The lowest value of the correlation coefficient, for the entire analyzed interval, is obtained for the smallest size of emitter rendered area (*d* = 6.4 nm). However this value continues to increase at the end of the interval. This means that we there are still small-scale details of the image, which could be potentially recovered, if we have recorded an infinite number of emitters. The scale of recovered details will be estimated, once we analyze the phase agreement in the frequency space. Figure 4 shows the correlation coefficient as a function of *d* for different number of emitters. We observe that each of these functions (except two functions representing a small number of emitters) has a local maximum in the analyzed interval. The position of such a maximum determines an optimal value of *d* used to render the emitters in the reconstructed image. Figure 5 shows the reconstruction of the ROI for different values of *d* and emitter density. We observe directly, that we need to choose a different optimal value of *d* for different number of collected emitters. 

**Fig. 6.**
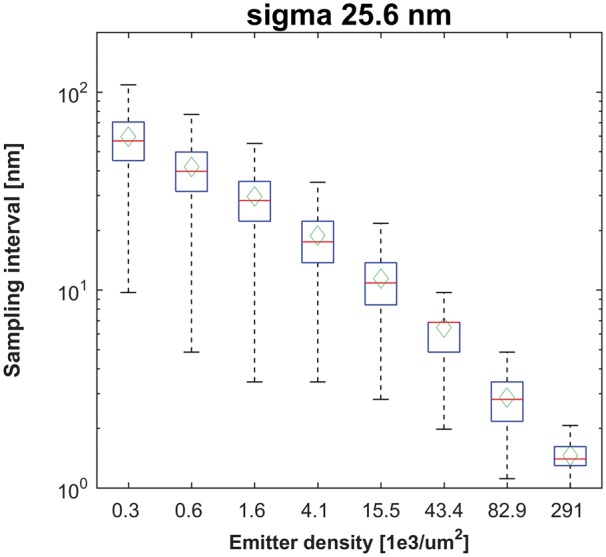
Distribution of (emitter) sampling density. Histograms of the radii of the mean area (δ*r*) are represented with mean (green diamonds), medians (red lines), 25/75th percentiles (blue boxes) and 5/95th percentiles (black whiskers) (Color version of this figure is available at *Bioinformatics* online.)

**Fig. 7.**
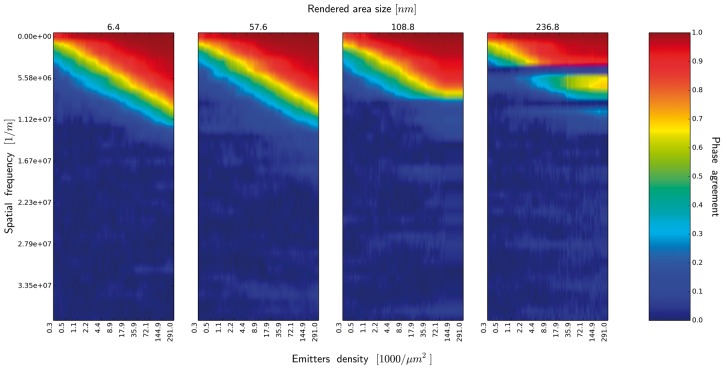
Similarity measure in frequency space, (image of neuronal tissue), emitter localization accuracy 25 nm. The spectrum of similarity measure is shown as a function of emitters density, for different rendered area size (side of square rendering). Only the lowest 1/3 of the function with the sampling resulting from the reference image resolution has been displayed

Subsequently, we analyze, whether for a specific theoretical resolution, we are able to reconstruct the corresponding fine scale image details. From Figure 4 we notice, that for the emitters density $∼10\cdot 10^{3}{\mu m}^{-2}$ the optimal value of the correlation coefficient is achieved, once we render the molecule position with an area of a square with side 57–108 nm. This value is ∼10× larger than the average sampling density (see Fig. 6) and ∼2× larger than the value obtained from the resolution test (see Supplementary Figs S9 and S10). Therefore, we conclude, that even with the very optimistic assumption (emitters density $∼10\cdot 10^{3}{\mu m}^{-2}$), our sampling density is still not sufficient to reconstruct the desired fine scale details. These observations are compatible with results reported by other groups (Nieuwenhuizen *et al.*, 2013), where resolution was limited by imaging density. This also means, that we are unable to extract the details at the Nyquist frequency, as shown by Figure 7, i.e. the phase agreement function in the frequency space (see Fig. 7). We notice immediately that there in no difference between the phase agreement function for d=6.4 nm and d=57.6 nm (and the intermediate values, not show). This is the consequence of the fact, that by increasing *d* we do not increase the information content in the image. What we do is the increment of spatial rendering of the sparsely probed large scale structures, which are better represented in the spatial rendering, therefore improving the possibility of quantitative measurements and visual interpretation of the image. Increasing the value of *d* to 108.8 nm we notice the deterioration of the phase agreement function, once we collected a certain number of emitters. This is the consequence of the fact, that we already started to reconstruct the fine-scale details smaller than the size of the emitter rendering area. This phenomenon is more prominent with d=211.2 nm when we started to introduce the aliasing artifacts, seen as a bands in the phase agreement function. Moreover, we observe that even in the most optimistic case we failed to reconstruct most of the fine-scale details. 

**Fig. 8.**
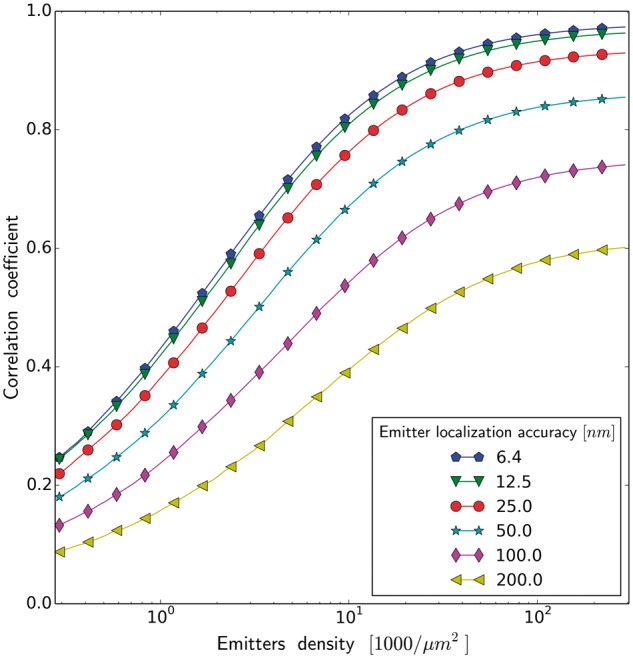
Correlation coefficient as a function of emitters density for different emitter localization accuracy (square rendering) (image of neuronal tissue)

**Fig. 9.**
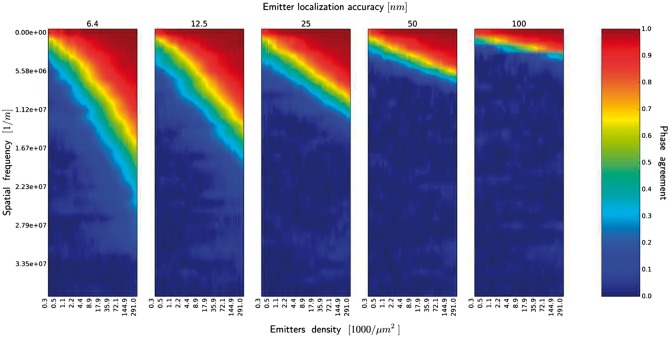
Similarity measure in frequency space, emitters rendered with squares with side *d* = 32 nm (image of neuronal tissue). The spectrum of similarity measure is shown as a function of emitters density, for different emitters localization accuracy. Only the lowest 1/3 of the function with the sampling resulting from the reference image resolution has been displayed

Next, we analyze an influence of accuracy of emitter localization (κ) on the quality of the rendered image. This accuracy is affected by the signal to noise ratio and pixel size. Figure 8 shows quantitatively to which extent the lack of fidelity of the reconstructed image is a result of the poor sampling or an inaccuracy in determining the position of emitters. The blue curve (κ=6.4 nm, i.e. the localization accuracy is equal to the resolution of the reference image) represents the limiting case, when the lack of fidelity of the resulting image is exclusively a consequence of a sampling process. With a poorer emitter localization accuracy we need clearly a larger number of emitters, to achieve the same quality of rendered image, note the logarithmic x− scale. Figure 9 shows the phase agreement functions for different localization accuracy. Even in the limiting case, κ=6.4 nm, and with the maximal density of emitters used in the simulations, we still failed to reconstruct most of the fine-scale details of the reference image. Note that we displayed only the lowest 1/3 of the function has been displayed (1/3 of the maximal frequency resulting from the reference image resolution). One might note that, with the same emitter densities, differences in reconstruction fidelity of the two kinds of biological structures (CN and NT) were observable. The image comprised few dense structures (regions of concentrated chromatin), which occupied small fraction of total image area while the major fraction corresponded to sparse labeling. Structures in the NT images (lipid membranes) were repetitive and distributed more uniformly. Therefore, more uniform sampling in the latter case produced better recovery of high spatial frequencies. 

Finally, we compare different types of area rendering (a square with side *d* versus Gaussian with 2*σ=d). We did not observe substantial differences in the values of quantitative parameters (see Supplementary Fig. S10).

## 4 Conclusions

In the presented article we quantified the fidelity of the reconstruction process with a STORM/PALM technique.

The numerical results show that, for the complex biological structures, the major limitation on image reconstruction fidelity is labeling density, rather than the localization precision. In other words, an amount of recovered information is a function of the sampling density in the first place, and a function of the localization precision, in the second place. Even for the unrealistically high sampling density, we still operate in the ‘labeling limited’ regime (Nieuwenhuizen *et al.*, 2013). Therefore even under the most favorable conditions, we were still unable to reconstruct accurately the observed biological objects, because our sampling was too sparse. These observations suggest that (i) we can improve an image quality by collecting more emitters, even with lower precision than the precision corresponding to the desired image resolution. We also find (ii), that since we are more restricted by sampling, than by precision, it pays off to reconstruct an image with a lower precision (larger rendered area) than the localization precision (see Fig. 4). The consequences (i) and (ii) mostly result from the fact, that sampling density is a local parameter affected by the architecture of the labeled biological structure. Thus, an ‘a priori model’ of the investigated structure (e.g. a synapse) may help to isolate the relevant features from the image and obtain corresponding biological information on the structure (e.g. on the receptor density).

Further, we notice, that the maximal spatial frequency is restricted by a minimal, rather than average sampling. In practical situation, we need to collect substantially larger number of emitters, than a number estimated from the resolution test assuming uniform emitters distribution. Finally, we observe that agreement between the images in the frequency space is independent of the rendered area size (until we reach aliasing, which occurs when the rendered area size becomes comparable with reconstruction resolution, at this stage we start to loose image details).

## Supplementary Material

Supplementary Data
