# Supplementary material for: Quality of biological images, reconstructed using localization microscopy data
Source: Bioinformatics. 2017 Sep 25;34(5):845–52. doi: 10.1093/bioinformatics/btx597 (PMC6192211; doi:10.1093/bioinformatics/btx597)
Supplement: Supplementary Data [file btx597_supplementary.pdf]

Supplementary information (SI):Quality of biological images, reconstructed using localization microscopy data

Blazej Ruszczycki and Tytus Bernas

September 6, 2017

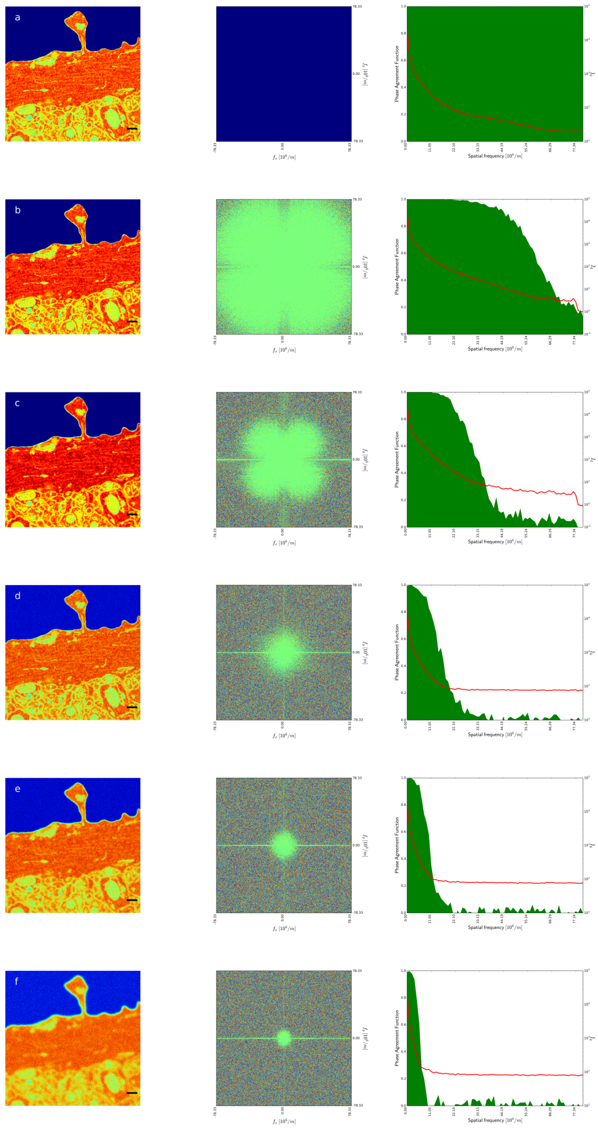

Figure S1: Similarity measure in frequency space for different image distortion; Left column- distorted image; Central Column- phase agreement between the distorted image and the original image $(\eta_0(f_x, f_y)\eta^*_{rec}(f_x, f_y))$; Right Column- Similarity measure $Q_{freq}(f)$ in frequency space (shaded green area, left scale), frequency spectrum (red line, right scale); a) Test image after Gaussian blur ($\sigma = 6.4\,nm$); b) Test image after Gaussian blur ($\sigma = 12.8\,nm$); c) Test image after Gaussian blur ($\sigma = 12.8\,nm$) with added noise; d) Test image after Gaussian blur ($\sigma = 25.6\,nm$) with added noise; d) Test image after Gaussian blur ($\sigma = 51.2\,nm$) with added noise; scale bars$= 500\,nm$

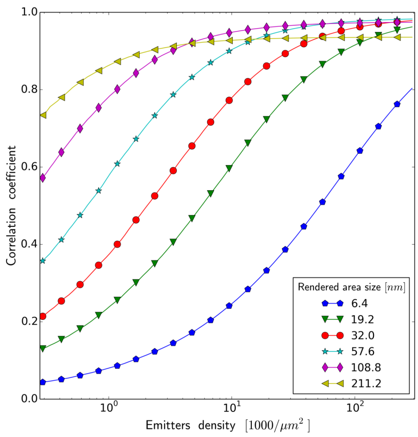

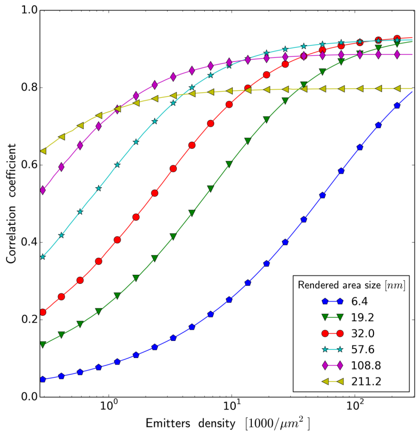

Figure S2: Correlation coefficent as a function of emitters density for different rendered area size (side of square rendering), image CN (cellular nucleus)- top, NT (neuronal tissue)- bottom, emitter localization accuracy $25\ nm$

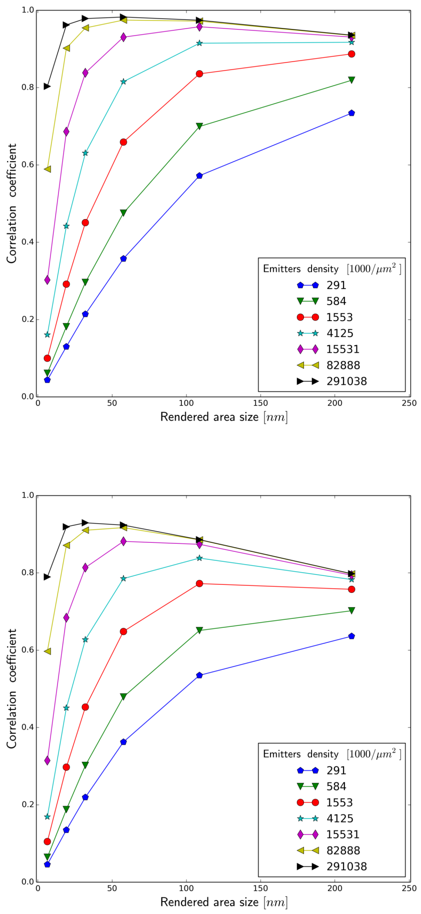

Figure S3: Correlation coefficient as a function of rendered area size (side of square rendering) for different emitters density, image CN (cellular nucleus)- top, NT (neuronal tissue)- bottom, emitter localization accuracy $25\ nm$

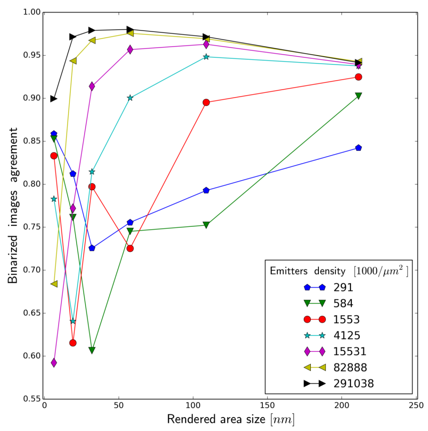

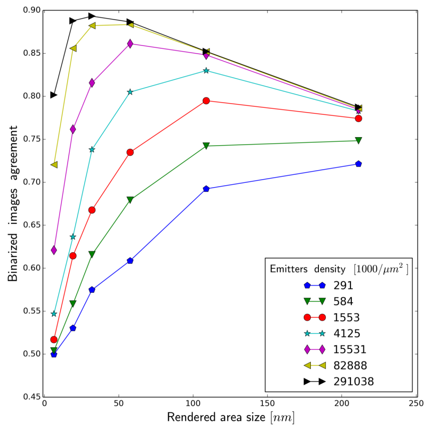

Figure S4: Agreement between binarized images as a function of emitters density for different rendered area size (side of square rendering), image CN (cellular nucleus)-top, NT (neuronal tissue)- bottom, emitter localization accuracy $25\ nm$

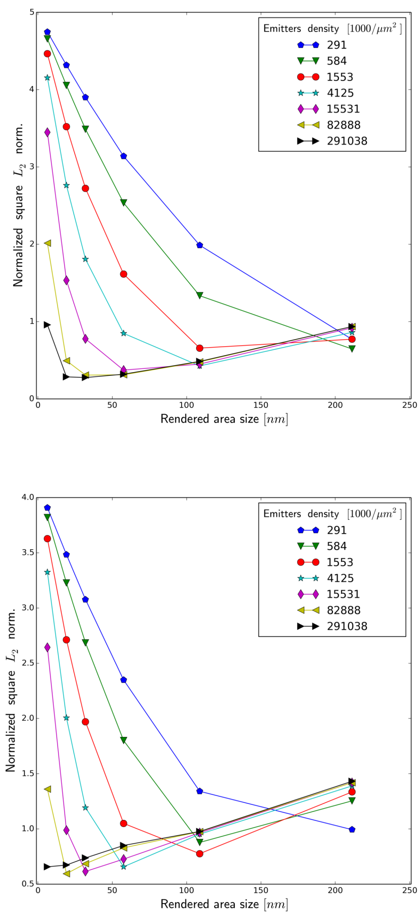

Figure S5: Normalized square L_2 norm as a function of emitters density for different rendered area size (side of square rendering), image CN (cellular nucleus)- top, NT (neuronal tissue)- bottom, emitter localization accuracy $25\ nm$

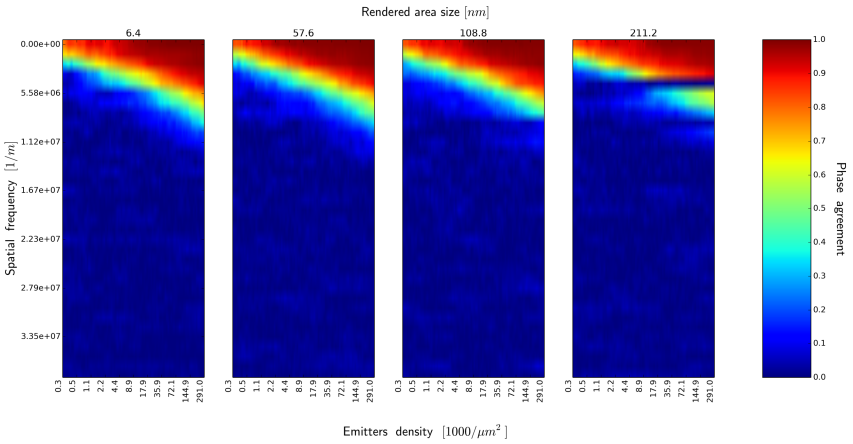

Figure S6: Similarity measure in frequency space, image CN (cellular nucleus)- top, NT (neuronal tissue)- bottom, emitter localization accuracy $25\ nm$. The spectrum of similarity measure is shown as a function of emitters density, for different rendered area size (side of square rendering). Only the lowest $1/3$ of the function with the sampling resulting from the reference image resolution has been displayed.

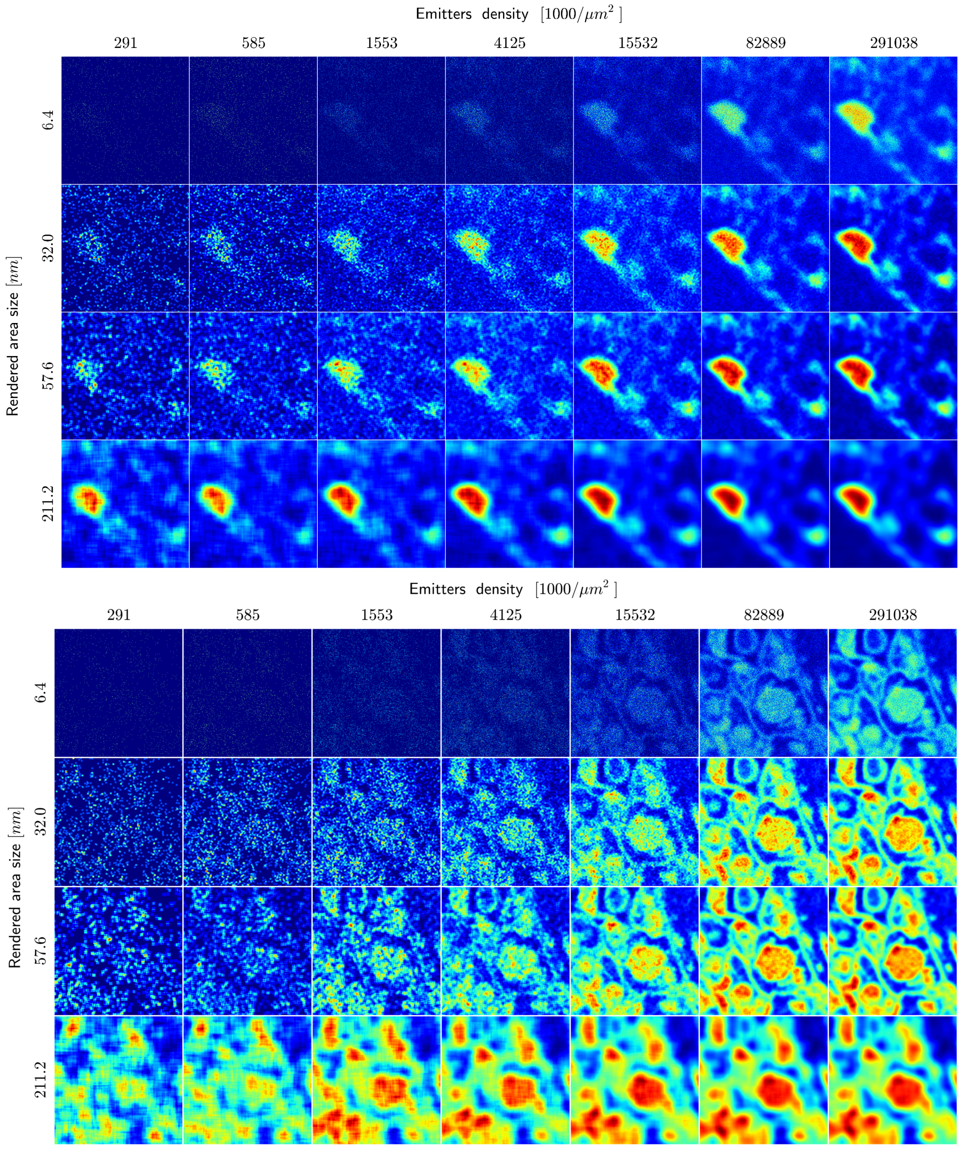

Figure S7: Reconstruction of the ROI for different rendering square size and emitter density, image CN (cellular nucleus)- top, NT (neuronal tissue)- bottom, emitter localization accuracy $25\mu m$

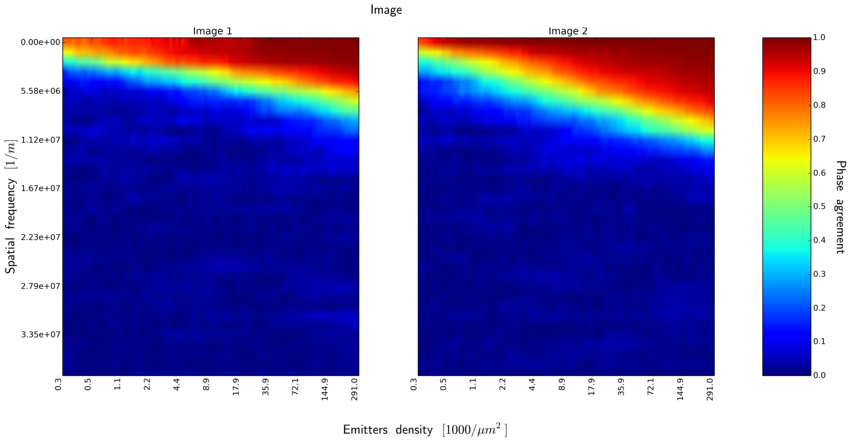

Figure S8: Similarity measure in frequency space, for different image modalities, CN (cellular nucleus)- left, NT (neuronal tissue)- right

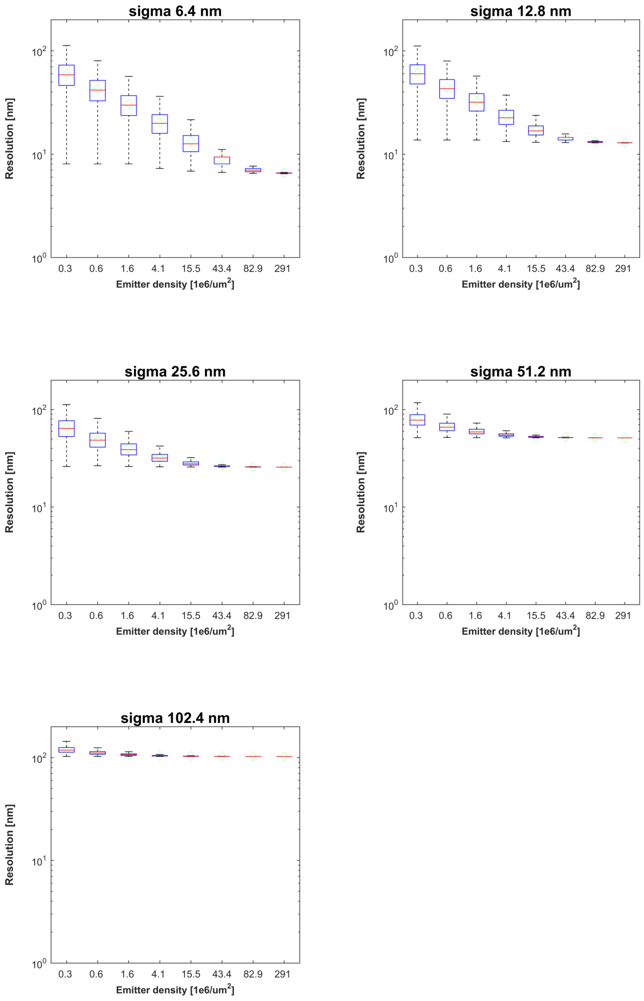

Figure S9: Resolution test for different values of σ. Histograms of the radii of the mean area (δr) are represented with mean (green diamonds), medians (red lines), 25/75 th percentiles (blue boxes) and 5/95 th percentiles (black whiskers)

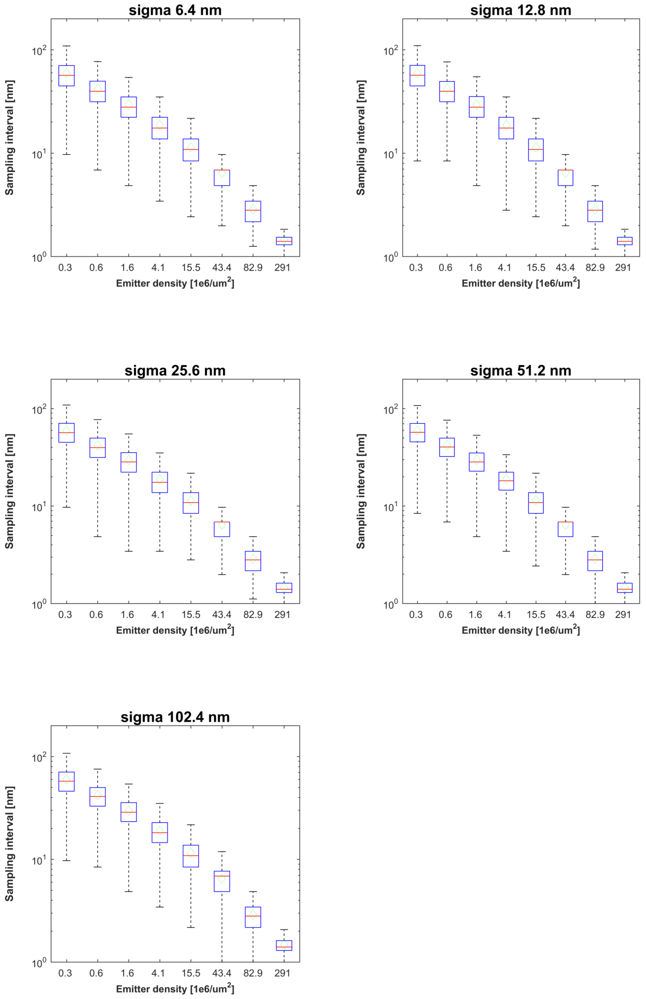

Figure S10: Distribution of (emitter) sampling density for different values of σ. Histograms of the radii of the mean area (δr) are represented with mean (green diamonds), medians (red lines), 25/75 th percentiles (blue boxes) and 5/95 th percentiles (black whiskers)

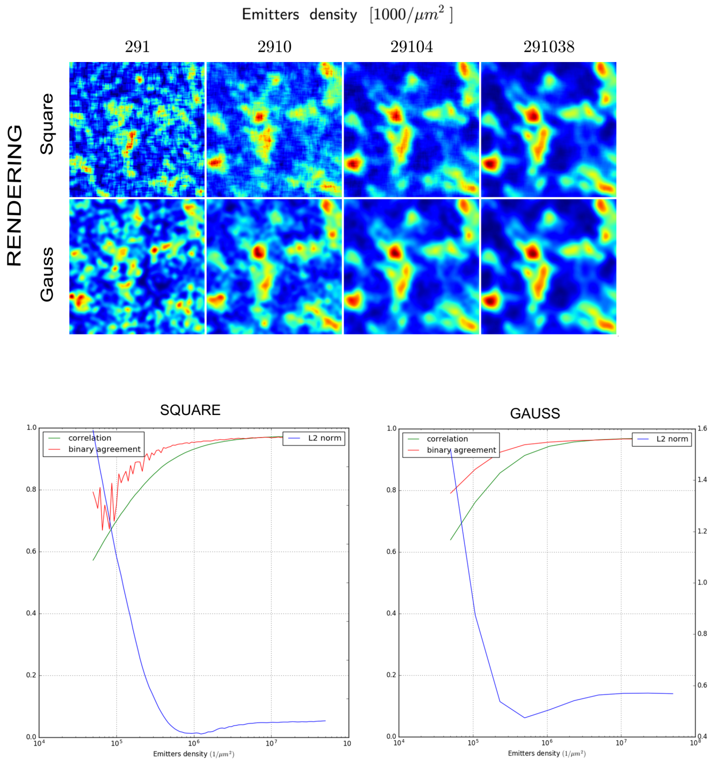

Figure S11: Comparison of rendering with a square area and Gaussian function. Top: reconstroction of ROI's. Bottom: Quantitative measures for different renderings
